# Usefulness of the Kinect‐V2 System for Determining the Global Gait Index to Assess Functional Recovery after Total Knee Arthroplasty

**DOI:** 10.1111/os.13547

**Published:** 2022-10-17

**Authors:** Hong Man Cho, Jangwon Seon, JiYeon Park, Jihoon Ahn, Young Lee

**Affiliations:** ^1^ Department of Orthopedic Surgery Gwangju Veterans Hospital Gwangju South Korea; ^2^ Veterans Medical Research Institute Veterans Health Service Medical Center Seoul South Korea

**Keywords:** Articular range of motion, Gait analysis, Knee arthroplasty

## Abstract

**Objective:**

The Korean Knee Society (KKS) score is used for functional evaluation during follow‐up after total knee arthroplasty (TKA), but it is time‐consuming to measure and is limited by its subjective nature. We investigated whether the global gait asymmetry index (GGA) that can be obtained using the Kinect‐V2 system could overcome the KKS limitations.

**Methods:**

Forty‐three patients who underwent TKA from January 2019 to December 2019 were included. Postoperatively, regular follow‐up was performed at 2, 4, 6, 8, and 12 weeks, and at 4, 6, and 12 months. At each follow‐up visit, the KKS was measured, and the walking path was followed with six Kinect‐V2 systems. After allowing the participants to walk naturally, the range of motion of each joint of the lower extremity and GGA were obtained. Changes in the KKS and GGA scores and measurement times were investigated until the final follow‐up. A statistical model was made to predict the KKS from the GGA score using data at all observed time points, and analysis of variance (ANOVA) with Turkey's post‐hoc tests and Pearson correlation tests were used for evaluation.

**Results:**

Both the KKS and GGA scores improved significantly from 4 weeks postoperatively until the final follow‐up. The measurement time was significantly shorter for the GGA (9.3 ± 1.4 min) than for the KKS (32.4 ± 9.2 min; *P* < 0.001) score. The predicted and actual KKS values clustered close to a straight line on the scatter plot, but the prediction was less accurate in the initial stage (2 weeks post‐surgery) than at later time points. The mean absolute error (MAE) and root mean square of the error (RMSE) were considered to be poorly predicted in the initial stage (8 weeks post‐surgery) compared to the later time‐points (MAE ≥ 5 and RMSE ≥ 6 for 8 weeks post‐surgery).

**Conclusion:**

In the early phase after knee joint surgery (up to 12 weeks post‐surgery), the GGA index does not predict the KKS well. However, after this time point, the GGA index can be simply measured in the outpatient department and may be able to replace the KKS. Thus, evaluation of the GGA index using the Kinect‐V2 may be a useful method to evaluate functional recovery in the outpatient clinic after knee joint surgery.

## Graphical abstract

Scatter plot of the actual and predicted Korean Knee Society (KKS) scores using the Global Gait Asymmetry (GGA) index model. The y‐axis is the actual KKS value, and the x‐axis is the KKS value predicted by the GGA index model. The values essentially cluster on a straight line, except that the preoperative and 2‐week postoperative points deviate markedly from the straight line.

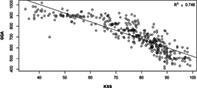

## Introduction

Total knee arthroplasty (TKA) has been recognized as a successful surgical procedure for treating knee arthritis that is refractory to conservative therapy. Although TKA is generally associated with favorable objective outcomes and high patient satisfaction,^1^ efforts have been made to improve objectivity in the clinical assessment of postoperative results by developing multiple scoring systems. Such objective treatment assessment has been performed using physical examination and radiologic assessment. However, physical examination and evaluation of pain and function have limitations in terms of the subjectivity of the examiner and the time‐consuming nature of completing many questionnaires. Various tools for functional evaluation after surgery are commonly used for the assessment of the knee joint; however, they may not be reliable for the assessment of the ability to perform high flexion in Korean patients who are expected to sit on the floor more often than westerners. In an attempt to overcome the limitations of these tools in assessing Korean patients with the floor‐sitting lifestyle, the Korean Knee Society devised the Korean Knee Society (KKS) score system designed to accommodate the evaluation of high flexion after TKA. As alternatives, kinematic analysis, muscle strength testing, and gait analysis were also performed during postoperative assessment.^2^ However, there are limitations to gait analysis because specialized equipment and a large space are required, and considerable effort is needed in its analysis. Among the methods of gait analysis, the method of acquiring 3D information using the kinematics approach is relatively difficult to handle and requires expensive devices. It is classified as marker tool based approach which analysis movement by sensors contacted to body and markerless tool based approach which assess movement by camera. Marker tool based approaches require many markers to be attached firmly to the body of the person. This process is time consuming and results in a cumbersome setup that can influence the naturalness of the motion. They are expensive, time consuming. For these reasons, in the last few years, considerable effort has been spent to study and implement markerless systems based on videography for gait analysis. Markerless video‐based approaches are less expensive; they are not cumbersome and do not affect the naturalness of motion, thus, they can be adopted to study human motion in an unconstrained environment. Lastly, they can be fully automatic and, hence, not operator dependent. The method using multiple kinematics cameras which is selected by authors is a state of the art system that provides a function to extract characteristic information such as position, speed and direction of an object to be observed through 3D motion assessment. Kinect 2 is markerless tool based approach 3D motion measurement device that can track and recognize the motion of object without the inconvenience of attaching a marker or holding an auxiliary device. Since the Kinect sensor can derive quantitative evaluation variables, it has the advantage of representing a standardized score for gait.

Here, we investigated whether it was possible to measure and analyze a patient's postoperative gait using a multi‐Kinect sensor‐based program, quantify this in terms of the Global Gait Asymmetry (GGA) index, and compare and analyze this with the existing KKS score as an objective evaluation method.^3^ Additionally, we investigated whether this approach would overcome the current difficulties of postoperative assessment, that is, the evaluator's subjectivity, the time and effort required for evaluation, and the need for large space or equipment.

## Materials and Methods

Of the patients who underwent outpatient evaluation after TKA between January 2019 and December 2019, and those who were available for a minimum follow‐up period of 1 year were retrospectively reviewed. Participants were excluded if they: (i) were unable to walk 10 m without a walking aid; and (ii) were unable to give informed consent, had rheumatoid arthritis, or had an unrelated musculoskeletal, neurological, or visual condition that might affect their movement. Cases with bilateral TKA, suspected postoperative infection, and cognitive inability to understand the questionnaire were also excluded.

The total number of patients was 43 (male: 23, female: 20; 43 knees) with a mean age of 73 years (range, 57–87 years). The operated side was the right in 21 knees and the left side in 22 knees. The preoperative diagnosis was degenerative osteoarthritis in 38 knees and post‐traumatic osteoarthritis in five knees. All operations were performed through the midvastus approach by a single surgeon (CHM) using the same technique. All prostheses were implanted with bone cement, and in all cases, a Triathlon implant (Stryker Orthopedics, Mahwah, NJ, USA) was used (Table 1). Rehabilitation was started with continuous passive motion and active dangling exercises on the first postoperative day. Partial weight‐bearing using crutches or a walker was allowed on the second postoperative day. Discharge was recommended on the 7th postoperative day. For 12 months postoperatively, follow‐up was performed at intervals of 2 weeks: 2, 4, 6, 8, and 12 weeks, and then at 4, 6, and 12 months.

**TABLE 1:** Demographic data of the patients

Total cases		43
Male: female		23: 20
Age (years)		73 (57–87)
Right: left		21: 22
Diagnosis	Degenerative osteoarthritis	38
	Post‐traumatic osteoarthritis	5
Approach	Mid‐vastus approach	43
Implants	Triathlon (stryker)	43

This retrospective cohort study was approved by the Clinical Trials and Biomedical Ethics Committee of our institution (VMC.Gwangju.19–15‐1). The requirement for written informed consent was waived because all patients provided consent for their anonymized clinical data to be analyzed and published for research purposes, upon admission.

### Postoperative Assessments Using the KKS

Clinical postoperative assessments were performed using the KKS clinical rating system.^3^ The KKS is composed of four subdomains (41 items): (i) pain and symptoms (12 items); (ii) function (17 items); (iii) evaluation of floor life (six items); and (iv) socio‐emotional function (six items). Each item can be scored up to 4 points, and the total score is converted to a 100‐point scale for evaluation.^3^ The evaluation was performed before surgery and at every follow‐up visit by the same orthopedic nurse unrelated to the surgery, and each KKS subdomain was evaluated. The evaluation time was measured from the start to the completion of the evaluation at each visit.

### Installation of Multiple Kinect Sensors

**Fig. 1:**
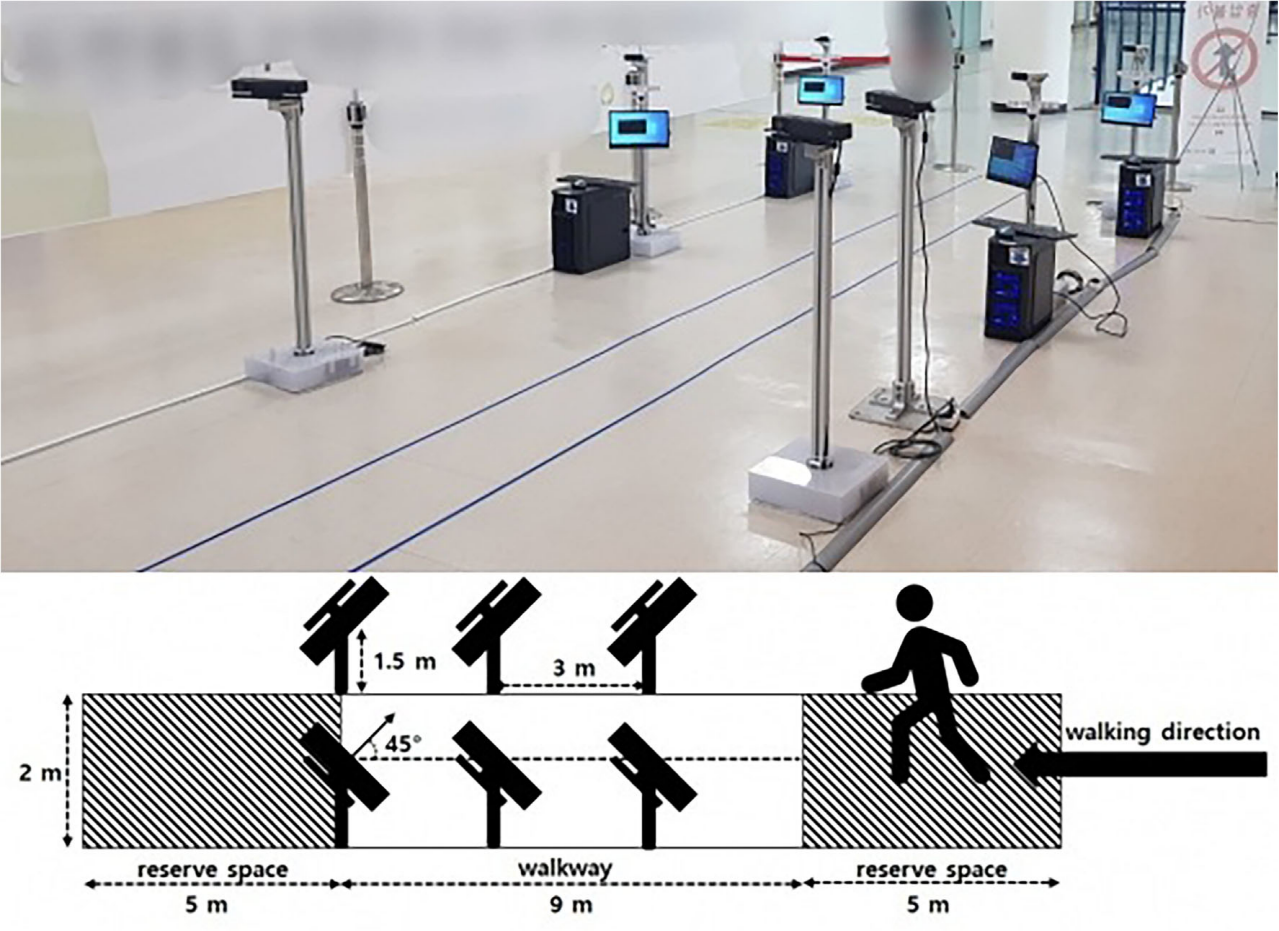
Overview of the multi‐Kinect‐V2 set‐up

Kinect‐V2 (Microsoft, Seattle, WA, USA) sensors were used to acquire the patient's surface image through an RGB‐D sensor that measured the time of flight after emitting infrared rays. Six Kinect sensors were arranged as an avenue (the multi‐Kinect‐V2 set‐up is displayed in Fig. 1). We placed the sensors pairwise in rows along the walking direction to reduce the self‐occlusion of the tracked person and to ensure more accurate tracking. A 9‐meter walkway was created, with three Kinect‐V2s installed on each of the left and right of the walkway, at intervals of 3 m. The system was installed at a vertical distance of 1.5 m from the ground and a distance of 2 m from the walking path to maintain the objectivity of measurement and configure the optimal measurement environment for body recognition using the depth sensor‐based motion analysis system. The orientation of the device was 45° in the walking direction. A 5‐m reserve space was placed at the beginning and end of each 9‐m walk. Before walking, calibration was performed using markers and 3D images generated by each Kinect within the visible area of all Kinect cameras.

After walking on the reserve space for 5 m, patients entered the walkway, and after their gait was measured for 9 m, they walked an additional 5 m and naturally stopped walking (totaling 19 m of walking) without the use of walking aids. Each patient walked the path 10 times, during which gait information was recorded.

### Gathering Gait Information Using Multiple Kinect‐V2 Sensors

**Fig. 2:**
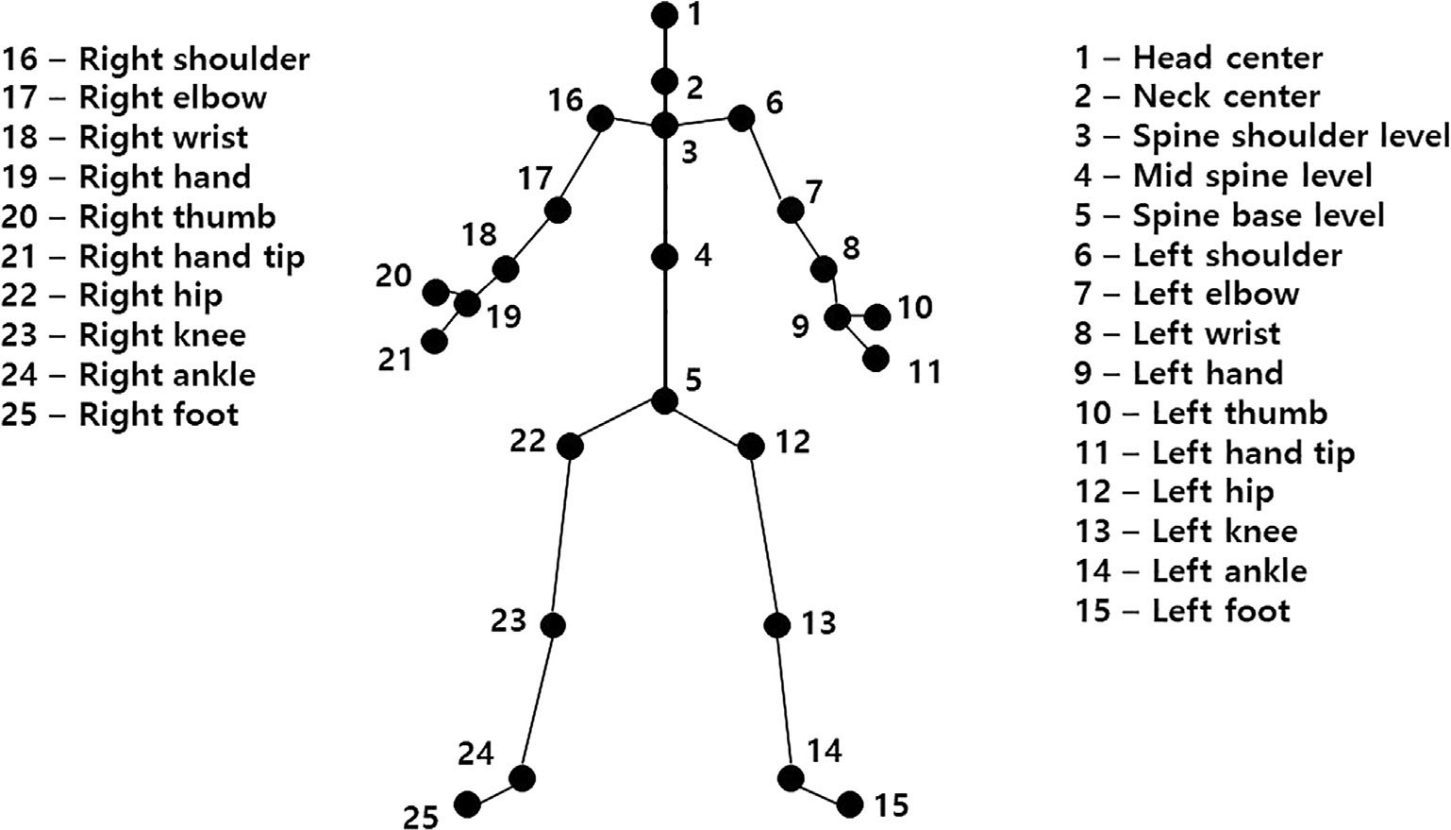
Body point determination with the human‐posture estimation software of Kinect‐V2 sensors

The Kinect for Windows Software Development Kit (SDK 2.0, www.microsoft.com) provides the 3D positions of 25 body points at a sampling rate of 30 Hz (Fig. 2). These body points are the head, neck, spine at shoulder level, mid‐spine mid, spine base, right and left shoulder, elbow, wrist, hand, thumb, hand tip, hip, knee, ankle, and foot. The joint motion angle was measured using the vector joining two recognized joints. The vector connects the main joint positions recognized from the depth image input through the depth sensor. The joint rotation angle was measured by calculating the Z‐Y‐X Euler angle through three direction vectors orthogonal to each other, using the right/left coordinate through the joint position.

### GGA Index

**Fig. 3:**
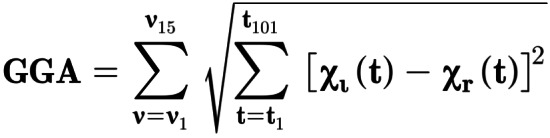
Calculation of the Global Gait Asymmetry index

The GGA index was used to evaluate gait symmetry. The GGA index was resolved into the joint coordinate system^4^ and filtered using a generalized, cross‐validatory quintic spline.^4^ The 3D joint angles of the hip, knee, ankle, and trunk in relation to the pelvis and the pelvis angle were used to compute the GGA score. All variables were time‐normalized to the right and left gait cycle (from heel strike to ipsilateral heel strike) into 101 equally distributed points. For each cycle, the GGA score was calculated (Fig. 3),^4^ where GGA is the GGA score, *v* are the angular variables, *t* are the time‐normalized points, and *xl* and *xr* are the values obtained for the left and right sides, respectively. The GGA is the sum of the magnitudes of 15 difference vectors (between consecutive left heel strikes for the left variables and between consecutive right heel strikes for the right variables), each with 101 components (time‐normalized data points). The score was always positive. The minimum obtainable value is zero, which represents perfect symmetry, and the score increases indefinitely with greater degrees of asymmetry. The GGA score was calculated for 10 right and left consecutive cycles for each condition and was averaged for each condition.

### Statistical Analysis

Data are expressed as the mean ± SD for continuous variables and as n (%) for categorical variables. Changes in KKS and GGA scores with time were evaluated using repeated‐measures analysis of variance (ANOVA) with Tukey's post‐hoc tests. The relationship between KKS and GGA at each time‐point was confirmed using Pearson correlation tests. A statistical model was developed to predict KKS in GGA from the data of all observed time‐points, using the generalized least‐squares method. The explanatory variables used in the model were sex, age, loc, time (each measurement point was considered as a continuous variable), (time)^2^, GGA, and (GGA)^2^. All statistical analyses were performed using the R software, version 4.0.1 (R Foundation for Statistical Computing, Vienna, Austria; http://www.R-project.org). A *P*‐value < 0.05 was considered statistically significant.

## Results

### KKS

**Fig. 4:**
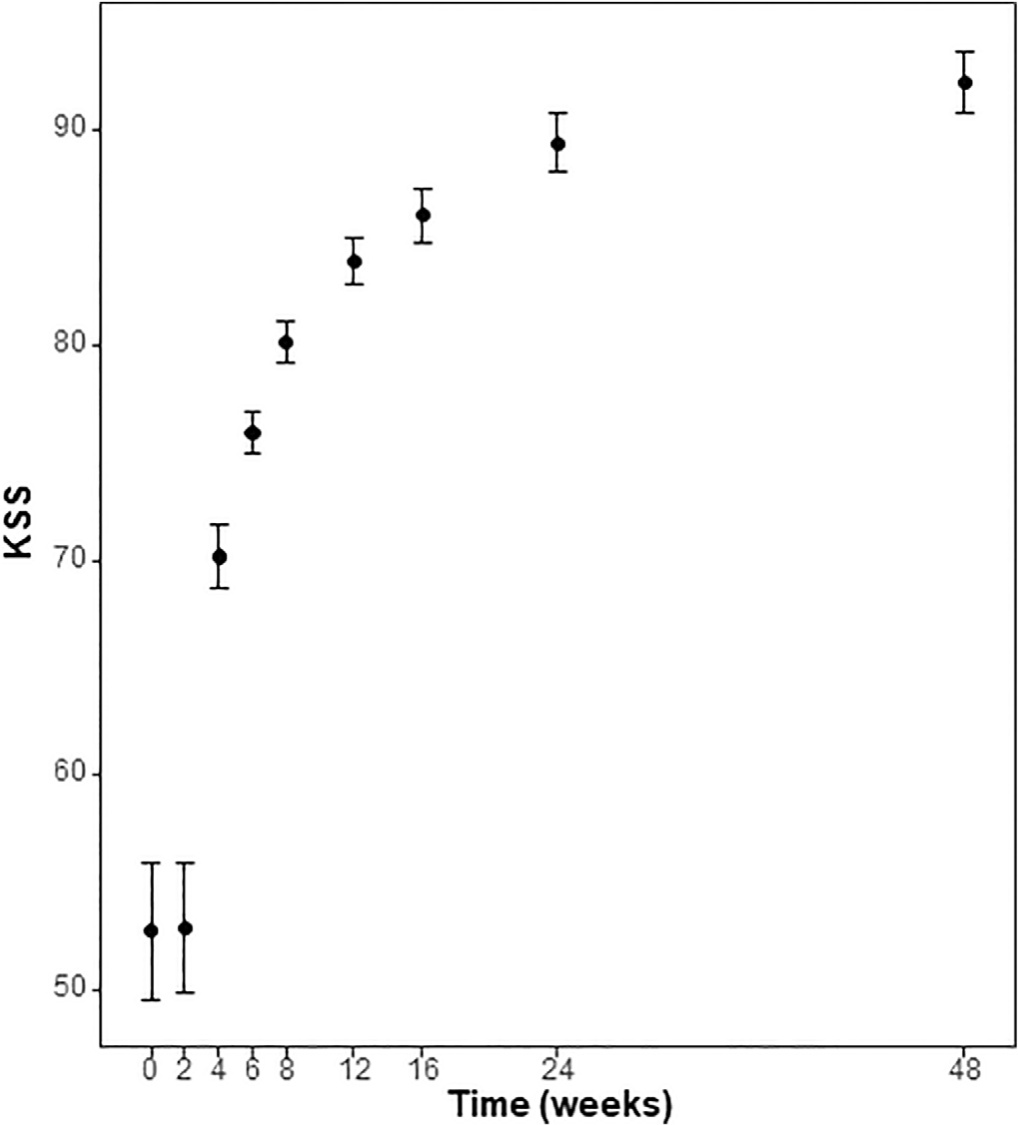
Overview of the analysis of change in the Korean Knee Society score (KKS) after surgery until the last follow‐up at 12 months post‐surgery. The average KKS for each time point is shown, and the error bar indicates the 95% confidence interval

The patients' preoperative KKS was 52.78 ± 10.39 and 52.96 ± 9.84 at 2 weeks, 70.19 ± 4.69 at 4 weeks, 75.94 ± 3.26 at 6 weeks, 80.11 ± 3.21 at 8 weeks, 83.92 ± 3.63 at 12 weeks, 86.04 ± 4.00 at 4 months, 89.37 ± 4.45 at 6 months, and 92.21 ± 4.77 at 12 months post‐surgery. There was no difference between preoperative and 2‐week postoperative values (*P* > 0.99), but there was a significant improvement compared to the preoperative values at all time points thereafter (*P* < 0.001) (Table 2; Fig. 4).

**TABLE 2:** Korean Knee Society (KKS) score and Global Gait Asymmetry (GGA) index measurement changes during the follow‐up period

Total		n = 43	
Sex	Male	23 (53.49%)	
	Female	20 (46.51%)	
Age		73.14 ± 7.53	
Loc	Right	21 (48.84%)	
	Left	22 (51.16%)	
		KKS	GGA
Required time		32.4 ± 9.2 min	9.3 ± 1.4 min
Preop score		52.78 ± 10.39	893.80 ± 47.29
Postop	2 wks	52.96 ± 9.84	899.16 ± 41.93
	4 wks	70.19 ± 4.69	817.37 ± 43.49
	6 wks	75.94 ± 3.26	765.39 ± 40.89
	8 wks	80.11 ± 3.21	705.21 ± 38.39
	12 wks	83.92 ± 3.63	661.85 ± 42.78
	4 mo	86.04 ± 4.00	613.81 ± 51.08
	6 mo	89.37 ± 4.45	570.16 ± 49.37
	12 mo	92.21 ± 4.77	505.80 ± 54.99

Abbreviations: mo, months; wks, weeks.

### GGA Scores

**Fig. 5:**
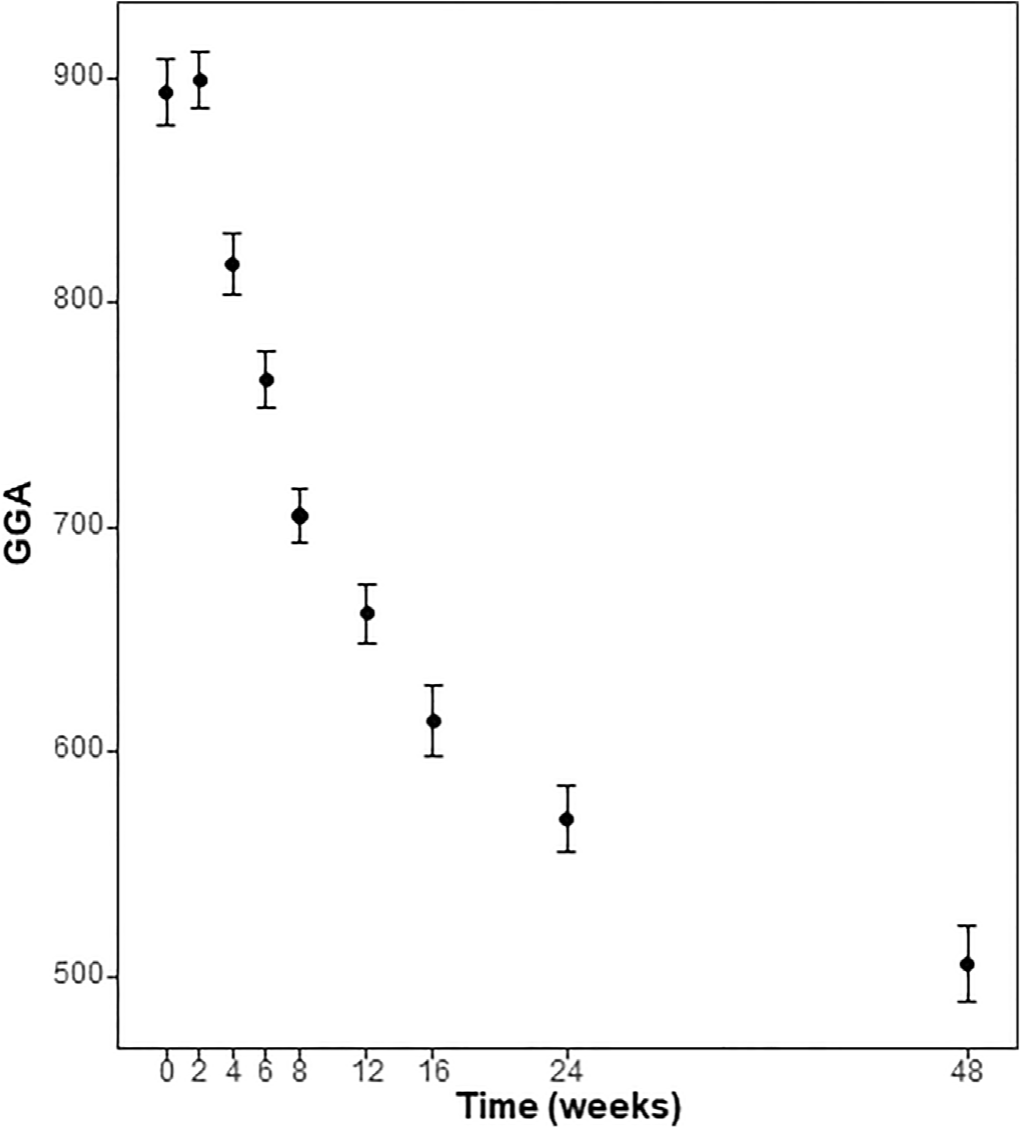
Overview of the analysis of change of the Global Gait Asymmetry (GGA) index after surgery until the last follow‐up at 12 months post‐surgery. The average GGA value for each time point is shown, and the error bar indicates the 95% confidence interval.

The patients' preoperative GGA was 893.80 ± 47.29. Postoperatively, GGA was 899.16 ± 41.93 at 2 weeks, 817.37 ± 43.49 at 4 weeks, 765.39 ± 40.89 at 6 weeks, 705.21 ± 38.39 at 8 weeks, 661.85 ± 42.78 at 12 weeks, 613.81 ± 51.08 at 4 months, 570.16 ± 49.37 at 6 months, and 505.80 ± 54.99 at 12 months post‐surgery. There was no difference between the preoperative and the 2‐week postoperative values (*P* > 0.99), but there was a significant improvement compared to before surgery at all time points thereafter (*P* < 0.001), similar to the KKS changes (Table 3; Fig. 5).

**TABLE 3:** Pearson's correlations between the Korean Knee Society score (KKS) and Global Gait Asymmetry (GGA) index at each time‐point

Time (weeks)	Correlation	*p* value
0	−0.196	0.208
2	−0.520	0.000
4	0.053	0.734
6	0.072	0.648
8	0.103	0.510
12	−0.202	0.195
16	−0.257	0.096
24	−0.379	0.012
48	−0.440	0.003

### KKS and GGA Score Measurement Time

It took 32.4 ± 9.2 min to measure the KKS (from the beginning of the first question to the time the last question was answered) (Table 2). The time required for GGA measurement (10 round trips to the spare space created at the beginning and end of the gait measurement space) was 9.3 ± 1.4 min (Table 3). Thus, the measurement time was significantly shorter for the GGA index (*P* < 0.001). KKS Repeated‐measures ANOVA showed that both the KKS and GGA scores differed significantly over time (*P* < 0.001). Compared by time‐point, there was no significant difference in both the KKS and GGA scores from before to 2 weeks postoperatively, but both KKS and GGA scores showed significant changes compared to preoperatively at all later time points (Table 2).

The relationship between the KKS and GGA scores at each follow‐up time‐point was confirmed using Pearson's correlation coefficient (Table 3). This correlation analysis of KKS and GGA at each point showed that high KKS at 2 weeks, 6 months, and 12 months postoperatively were significantly negatively correlated with GGA scores, but the correlation was not significant at any other time points.

**Fig. 6:**
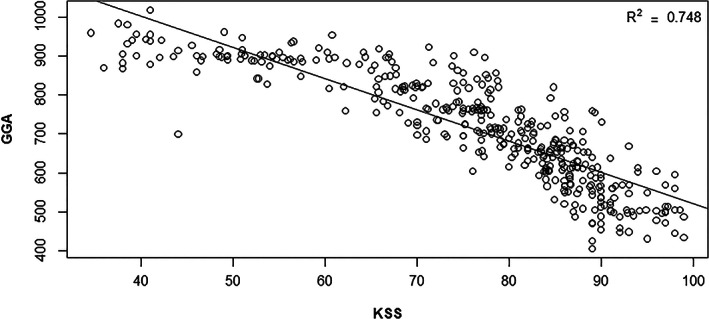
Scatter plot of actual and predicted Korean Knee Society (KKS) scores using the Global Gait Asymmetry (GGA) index model. y‐axis, actual KKS value; x‐axis, KKS value predicted by the GGA index model. Values essentially cluster on a straight line, except that the preoperative and 2‐week postoperative points deviate markedly from the straight line

We created a model that predicted KKS from the GGA index, using data from all observed time points. This model predicted KKS from sex, age, loc, time (considering each measurement point continuously), (time),^2^ GGA, and (GGA)^2^ variables, using the equation KKS = 38.84–0.53 × sex +0.01 × age ‐ 0.68 × loc + 1.57 × time ‐ 0.02 × (time)^2^ + 0.10 × GGA ‐ 8.72 × 10^(−5)^ × (GGA)^2^ (Table 4). To determine how well GGA can predict the KKS, we obtained the predicted KKS value based on this model equation and plotted the actual and predicted values on a scatterplot. Mean absolute error (MAE) and root mean square of the error (RMSE) were calculated at each point. The predicted and actual KKS generally clustered close to a straight line, but the predicted KKS value was less accurate at the initial time‐point (2 weeks post‐surgery) than at subsequent time points. This implied that the GGA index cannot predict the KKS in the early stages postoperatively but improves thereafter (from 4 weeks post‐surgery) (Fig. 6). Although there is no absolute criterion for MAE and RMSE, the closer these values are to 0, the better the prediction is considered to be. For the “before vs. 2 weeks post‐surgery” comparison, the MAE was ≥8 and RMSE was ≥10, indicating relatively poor prediction as compared to other time points. Even at the 8th week, the MAE was ≥5 and RMSE was ≥6, which indicated a relatively poor prediction as compared to subsequent time points. From the 12th week onwards, the MAE and RMSE were ≤6, indicating a relatively better prediction than before the 12th week (Table 5).

**TABLE 4:** Results of the estimated KKS (Korean Knee Society score) prediction model using a generalized least‐square method (LOC 1: right 2: left knee)

	Beta	SE	95% CI lower bound	95% CI upper bound	*P* value
(Intercept)	38.84	7.23	24.67	53.01	0.000
Sex	−0.53	0.66	−1.82	0.77	0.426
Age	0.01	0.04	−0.08	0.10	0.790
Loc	−0.68	0.67	−1.99	0.62	0.307
Time	1.57	0.10	1.36	1.77	0.000
I (time^2^)	−0.02	0.00	−0.02	−0.02	0.000
GGA	0.10	0.02	0.06	0.14	0.000
I (gga^2^)	−8.72E‐05	1.49E‐05	−1.16E‐04	−5.80E‐05	0.000

Abbreviations: CI, confidence interval; SE, standard error.

**TABLE 5:** Accuracy of KKS (Korean Knee Society score) prediction

Time (weeks)	MAE	RMSE
0	8.83	10.38
2	8.42	10.54
4	5.21	6.63
6	5.49	6.40
8	5.32	6.17
12	4.24	4.85
16	2.77	3.91
24	3.86	4.82
48	4.11	5.36

*Note*: For the mean absolute error (MAE) and root mean square of the error (RMSE), a value closer to 0 indicates better prediction.

## Discussion

In this study, we investigated whether the GGA index that can be obtained using the Kinect‐V2 system could overcome the current KKS limitations. We showed that, in the early stages post‐TKA, the GGA index might not be able to replace the KKS, as the correlation remained low until 12‐weeks post‐surgery. However, after this time‐point, the GGA index can be measured during outpatient follow‐up using a Kinect‐V2 set‐up and may be able to replace the KKS. Thus, evaluating the GGA index using the Kinect‐V2 may be useful for evaluating functional recovery in the outpatient clinic post‐TKA. We did not use a 3D motion capture system or wearable IDEEA® Life Gait System because the research aim of this study was to collect a wide range of general data considering the minimum space, effort, measurement time, and cost. However, if a system such as a 3D motion capture system or wearable IDEEA® Life Gait System becomes simpler to wear and less expensive, and can be easily used to obtain measurement and evaluation results, it will be helpful for improving research in the future.

### Postoperative Functional Evaluation

Evaluation of TKA outcomes needs to be reproducible, based on objective parameters and should permit assessment of functional performance after TKA.^5^ Some studies have attempted to conduct an objective investigation based on the evaluation of kinematics, proprioception, quadriceps force, and alternative questionnaires.^6^, ^7^, ^8^ The KKS that we used is known to have eliminated ceiling effects compared to several other clinical rating systems, and, in the Asian population, the floor life subdomain of the KKS can be used as a predictor of postoperative knee flexion.^9^

### Functional Evaluation Using Gait Information

The assessment of functional recovery using gait analysis has been reported to be an objective and highly reproducible evaluation in numerous studies,^10^ but it has the disadvantages of requiring expensive equipment or large space, and there are many difficulties in its assessment and analysis, limiting its use.

### Analysis of Gait Information Using Kinetic Camera

Lee *et al*.^11^ reported that ROM measurement of a joint using a depth sensor‐based motion analysis system, such as the Kinect, had a high intraclass correlation coefficient and matched well to measurement using a protractor. As such, the depth sensor‐based motion analysis system using infrared sensors may overcome many shortcomings of conventional gait analysis methods, such as the relatively high economic cost and need for a large place, and several studies have reported the usefulness of gait analysis using the Kinect‐V2.^12^, ^13^, ^14^, ^15^ In measuring the ROM with the Kinect‐V2, we first sought to secure objectivity by utilizing the user's gait information in all Kinect cameras' visible areas before measurement, and by performing calibration of multiple Kinect sensors before the experiment using 3D images and markers generated by each Kinect. Second, depth sensor‐based motion analysis systems can be limited by noise values due to body part misrecognition when the measurement object shows fast movement. A measurement system that can predict an object's position with continuous movement after a certain period of time, and applies a Kalman filter algorithm that is resistant to noise, was therefore used in this study. Third, because the subject's surface image and joint position are measured through infrared rays, clothes made of polyester or black clothes reflecting infrared rays can act as obstacles to measurement; thus, if necessary, participants were asked to change to cotton clothes with low reflectivity.

### Importance of Gait Symmetry among Gait Information

Among the various information that can be obtained through gait analysis, gait symmetry is known to be important in measuring gait pattern alterations, establishing the level of functional limitation due to pathology, observing its changes over time, and evaluating the effects of rehabilitative intervention. The degree of gait asymmetry is important in attempting to restore function, and a target gait function is a useful outcome measure of rehabilitation.^16^ In the results of our study, both the KKS and GGA index showed significant improvement from 2 weeks after surgery, and similar results were obtained with previous studies indicating that the symmetry of both lower extremities can be used to evaluate functional recovery. The GGA index is not an absolute measure representing the symmetry of both lower extremities. However, with the development of future technology, if the symmetry of both sides can be measured more accurately using a relatively inexpensive kinetic camera in a small space, we consider that the GGA index could be used for functional evaluation after knee joint surgery.

Studies in the field of orthopedic surgery have investigated the recovery of function after hip joint replacement by analyzing the symmetry of walking,^17^, ^18^ and the feasibility and efficacy of incorporating symmetry training into the rehabilitation of total joint replacement patients have been reported.^19^ For many unilateral surgical procedures, such as lower limb ligament reconstructions or joint replacements, increasing gait symmetry is desirable.^19^, ^20^ The importance of achieving full symmetrical knee ROM, both pre‐ and post‐anterior cruciate ligament reconstruction, has been highlighted as the most important factor for ensuring the best long‐term successful surgery results and patient satisfaction.^20^ The GGA index measured with a Kinect is low cost, easy to use, and is a promising development for clinical gait analysis.^21^

### Significance of Gait Symmetry Evaluation Using Kinetic Camera

Therefore, we also attempted to determine the angle of the main joint using the Kinect‐V2 to evaluate gait symmetry, in order to assess functional recovery. Despite its clinical interest and importance, there are several limitations in measuring gait symmetry when using Kinect sensors.^16^ First, the Kinect‐V2 sensor has a horizontal field‐of‐view of approximately 70° and can reliably cover a 4.5‐m depth. Given the limited size of the tracking volume of the Kinect sensor, set‐ups with multiple Kinect sensors have been proposed for covering a larger volume.^13^ To address this issue, we used six Kinect cameras for gait analysis and paid attention to the spatiotemporal calibration of the system and sensor placement. Second, while achieving gait symmetry is an important aspect of rehabilitation after lower limb surgery to avoid long‐term unilateral loading, to date, the required level of symmetry is not known and is typically clinically evaluated by a patient's level of pain or discomfort due to their asymmetric gait. Furthermore, diagnoses based on standard clinical methods are subjective and influenced by physicians' and patients' perceptions.^22^ Third, there is currently no standardized method for measuring gait symmetry. Many indices in functional diagnostics are used to assess the degree of asymmetry: relative difference index,^23^ relative asymmetry index,^24^ symmetry angle,^25^ standard deviation of the differential index for upper limbs,^26^ and GGA score.^27^ The GAA score is reported to be sensitive to different asymmetry levels and may be useful for rehabilitation and assessment.^27^

### Comparison of KKS and Gait Symmetry Using Kinetic Camera (GGA Index Value)

We created a model that predicts the KKS from GGA index values, using data from all time points. Our results indicated that, in the early period after TKA (before 12 weeks), GGA may not be able to replace KKS, given the low predictive power at this time‐frame. However, from the 12th week after TKA, the GGA index can be simply measured in an outpatient clinic, and can be used to predict the KKS. Thus, this may be a useful method for evaluating functional recovery during the outpatient examination after knee joint surgery. We considered walking aids to be the cause of the low predictive value in the early period, until 12 weeks after surgery. Since KKS mainly consists of questions and answers, there is a possibility that patients using canes or crutches may give relatively positive answers. However, since the patients had to walk without walking aids in the gait measurement space created for this study, it could be that although the patients subjectively might have thought that their condition had improved, the actual gait did not reflect this. To further investigate this, we plan to conduct a prospective study by dividing the patients into a group evaluated with KKS after measuring gait, and a group evaluated with KKS and gait measurement.

The addition of stride length or gait velocity to the analysis, or a further increase in the number of cameras and data, may improve the model's predictability in the initial stage. Moreover, if data can be obtained by quantifying the positions of images and joints through programs that apply deep learning technology, in addition to calculating the rotation angle of the joint by calculating the Z‐Y‐X Euler angle through the three‐direction vectors, the predictability may be further improved.

Additionally, to create a prediction model, training and test sets should be created; after the model is generated based on the training set, its prediction results should be evaluated on the test set. However, in this study there may be an overfitting problem because the reported predictive model was created and evaluated without dividing the patients into training and test sets, due to the small sample size. Thus, more cases should be accumulated for future evaluation. Moreover, the reality and feasibility of GGA still need to be proven, and whether GGA can accurately reflect changes in joint function needs to be confirmed. Therefore, it is still inappropriate to directly associate two different parameters, such as KKS and GGA. In the future, it is necessary to investigate the changes in joint function in more detail through follow‐up data. Finally, in order to compare and verify the GGA index reported in this study, the accuracy and repeatability in more cases should be investigated; in these cases, both KKS and GGA measurements will be needed to more accurately evaluate recovery, because gait asymmetry evaluation of patients could not completely replace the KKS questionnaire.

### Strengths and Limitations

This was an early study investigating whether lower extremity function evaluation is possible based on symmetry assessment obtained through gait measurement. Combining additional information that can be obtained through gait measurement, other than symmetry, with various clinical evaluation results is expected to further improve the accuracy of the reported model in the future. In this study symmetry and specific formulas were used to analyze the information obtained through gait. However, there is a lot of information beyond symmetry that can be obtained through the evaluation of gait, which can be used to assess lower extremity function. Information that can be obtained by analyzing gait includes information in a continuous form and information expressed as a single mathematical number. Therefore, it is necessary to develop a tool that can evaluate the functional recovery of the lower extremities by analyzing this various and complex information as a whole. In addition, it should be possible to further reduce the enlargement and reduction of images that appear during shooting, and the deviation of camera measurements according to the walking direction. Such improvements are expected to lead in the acquisition of better data even during the early stages after surgery when gait is too unstable. These developments are expected to mitigate and overcome the limitations associated with the model reported in this study.

### Conclusion

Based on the findings of this study, it could not be confirmed that gait symmetry assessment and the reported GGA index obtained through kinetic cameras could completely replace KKS for evaluating functional recovery after TKA. In particular, the predictive value of the reported GGA index was low until 12 weeks after surgery; this could be attributed to incomplete recovery in the early stage after the operation, and to the gait being too unstable during that period. However, GGA and KSS improved in the same manner from 4 weeks after surgery onwards, and the predictive value of GGA for KSS improved from 12 weeks after surgery. Therefore, the proof of this concept study suggests that, with further improvements, it is possible that the reported GGA index could replace KSS for evaluating functional recovery after TKA. This can lead to a plethora of advantages, associated with low cost, ease of availiability and portability of the device, and decreased time required for evaluation of functional recovery after TKA.
